# Competition of Parental Genomes in Plant Hybrids

**DOI:** 10.3389/fpls.2020.00200

**Published:** 2020-02-25

**Authors:** Marek Glombik, Václav Bačovský, Roman Hobza, David Kopecký

**Affiliations:** ^1^Institute of Experimental Botany, Czech Academy of Sciences, Centre of the Region Hana for Biotechnological and Agricultural Research, Olomouc, Czechia; ^2^Department of Plant Developmental Genetics, Institute of Biophysics of the Czech Academy of Sciences, Brno, Czechia

**Keywords:** interspecific hybridization, genome stability, whole-genome duplication, allopolyploid, homoeologous recombination, chromosome pairing, fertility

## Abstract

Interspecific hybridization represents one of the main mechanisms of plant speciation. Merging of two genomes from different subspecies, species, or even genera is frequently accompanied by whole-genome duplication (WGD). Besides its evolutionary role, interspecific hybridization has also been successfully implemented in multiple breeding programs. Interspecific hybrids combine agronomic traits of two crop species or can be used to introgress specific loci of interests, such as those for resistance against abiotic or biotic stresses. The genomes of newly established interspecific hybrids (both allopolyploids and homoploids) undergo dramatic changes, including chromosome rearrangements, amplifications of tandem repeats, activation of mobile repetitive elements, and gene expression modifications. To ensure genome stability and proper transmission of chromosomes from both parental genomes into subsequent generations, allopolyploids often evolve mechanisms regulating chromosome pairing. Such regulatory systems allow only pairing of homologous chromosomes and hamper pairing of homoeologs. Despite such regulatory systems, several hybrid examples with frequent homoeologous chromosome pairing have been reported. These reports open a way for the replacement of one parental genome by the other. In this review, we provide an overview of the current knowledge of genomic changes in interspecific homoploid and allopolyploid hybrids, with strictly homologous pairing and with relaxed pairing of homoeologs.

## Introduction

Interspecific hybridization merges genomes from two different species or even genera. Compared to animals, interspecific hybridization is much more common in plants and significantly contributes to plant speciation. In fact, many backcross hybrids probably remain undetected as they may be indistinguishable from parental species (Mallet, 2005). In plants, interspecific hybridization is frequently accompanied by whole-genome duplication (WGD), which is only rarely observed in animals. There is evidence that all angiosperms have undergone at least one round of WGD during their evolutionary history (Jiao et al., 2011; Ruprecht et al., 2017), and it is estimated that 30–70% of extant plant species are polyploids (Masterson, 1994). Hybridization is frequently accompanied by enhanced heterozygosity and hybrid vigor (e.g., growth and seed production), while WGD restores the fertility of a newly formed hybrids and contributes to the stabilization of the hybrid genome, fixing both heterozygosity and new hybrid characters (Chen, 2010). Interspecific hybridization may also lead directly to speciation without polyploidization, but such homoploid hybrids are rare. To date, only a limited number of putative homoploid hybrid speciation events have been documented in flowering plants (Yakimowski and Rieseberg, 2014).

Apart from the evolutionary aspect of interspecific hybridization in plants, many major crops such as wheat, oilseed rape, banana, tobacco, coffee, and cotton also originated from hybridization of two or more species. Moreover, wide hybridization is frequently used in breeding programs to increase the global genetic diversity of the crop gene pool. This can be accomplished either by the creation of a new crop species, such as Triticale (hybrids of wheat and rye) and Festulolium (a hybrid of fescue and ryegrass) or by the introgression of specific loci from wild relative into the recipient crop. Allopolyploidy may generate intergenomic heterosis, which results in a competitive advantage over diploid progenitors (Comai, 2005), and it may mask deleterious recessive alleles and increase mutational robustness (Madlung, 2013). Newly formed hybrids often display broader adaptation to new environmental niches relative to their parents and may show greater ability to colonize disturbed and harsher habitats (Rieseberg et al., 2007; te Beest et al., 2012). This, in turn, may increase the invasiveness of newly formed hybrids (Pandit et al., 2011).

Despite the evolutionary success of allopolyploids, many newly developed hybrids display a phenomenon known as hybrid lethality. Dobzhansky (1936) proposed a model explaining the paradox of hybrid vigor (or evolutionary success) and incompatibility as the interactions of the parental genomes; this is now explained by the role of divergent small RNAs (Ha et al., 2009). New plant hybrids undergo multiple changes at the genome, chromosome, and gene levels. This includes genome downsizing, structural chromosome rearrangements, amplifications and/or reactivation of repetitive elements, modification of the gene expression patterns, and concerted evolution of multigene families (such as rDNAs immediately after the formation of the hybrid individuals). Furthermore, divergence of small RNAs in parental genomes may contribute to multiple heritable (epigenetic) changes, not associated with the changes in the DNA sequence (Bartel, 2004; Rao et al., 2009). The magnitude of all changes associated with hybridization is probably dependent on the degree of genome differences (Garsmeur et al., 2014; Bird et al., 2018).

**FIGURE 1:**
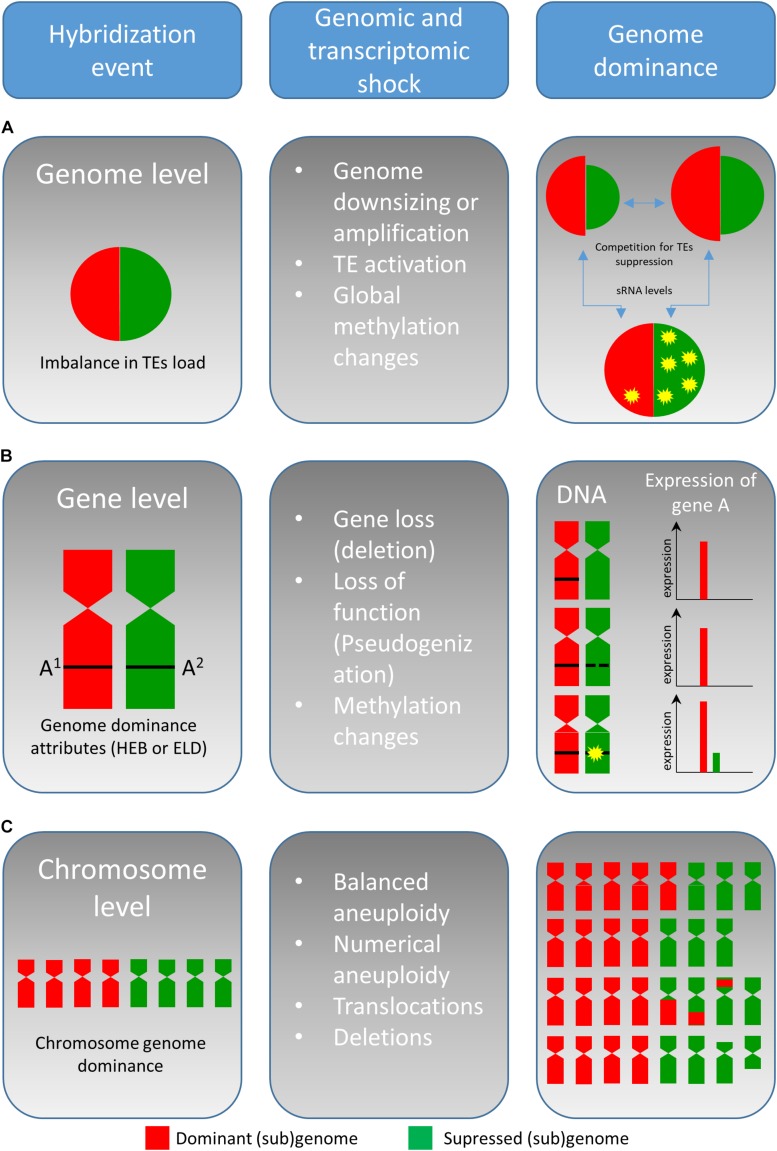
Simplified model on the genetic and epigenetic changes associated with the interspecific hybridization in plants. The genome dominance described in plant interspecific hybrids can act on genome, chromosome, and gene level. Hybridization event, genome size, and TEs activation is affected greatly by imbalance(s) in TEs load and overall level of sRNA. The imbalance results in (sub)genome competition for TEs suppression **(A)**. Soon, after hybridization, functional conflicts between interacting genes impair the expression and due to gene imbalance, one genome becomes to be dominant at the expression level. Attributes facilitating the dominance at the transcriptome level are expression level dominance (ELD) and homoeolog expression bias (HEB) **(B)**. At the chromosomal level, genome dominance is affected by several factors (listed in section “Parental Chromosome Dominance”) and by chromosomal aberrations, leading to imbalance in chromosome number **(C)**.

Allopolyploidization has been intensively studied since its discovery, and several outstanding review papers concerning various aspects of allopolyploidization have been published in recent years (Mallet, 2005; Soltis and Soltis, 2009, 2012; Schubert and Lysak, 2011; Bottani et al., 2018; Li et al., 2019). In this review, we endeavor to provide a different view focusing on the fusion of two parental genomes and their competition in the single nucleus. With an increasing number of reports on the hybrid genome structure and evolution, it is evident that one of the parental genomes becomes dominant, and the other is rather submissive/suppressed in successive generations. This phenomenon called “genome dominance” (sometimes referred as “subgenome dominance”) (Thomas et al., 2006) may include a plethora of the features including (a) an increase in genome size of the dominant genome and/or reduction of the submissive one, (b) elimination of chromosomes of the submissive genome, (c) replacement of chromosomes from the submissive genome by those of the dominant genome, (d) preferential loss (deletion) or silencing (by epigenetic processes) of alleles from the submissive genome resulting in homoeologous expression bias and the expression level dominance (ELD), and (e) preferential activation of transposable elements (TEs) and (f) global methylation changes (all these events are listed in Figure 1). Because the divergence in the mating system and parental conflict (acting as a barrier to hybridization) are discussed elsewhere (Brandvain and Haig, 2005), we will discuss the features of genome dominance at the level of genomic changes, chromosomal pairing, and gene regulation in relation to the stability of hybrid genomes.

## Genomic Changes and TE Dynamics in Allopolyploids

Genomic stress represented by interspecific hybridization (and polyploidization) affects genome reorganization, genetic changes, and epigenetic repatterning (histone modifications and DNA methylation). Global genomic changes go hand in hand with the establishment of genome dominance (Edger et al., 2017) and “new” gene expression, TEs reactivation, and TEs new insertions (Parisod et al., 2010b; Yaakov and Kashkush, 2012). Activation of TEs might occur immediately after a WGD event and seems to play a major role in all genomic changes in allopolyploids (Parisod et al., 2010a). In fact, the activation of TEs seems to be dependent on the qualitative/quantitative imbalance between the parental TE loads (Figure 1A). Such imbalance then may result in weak suppression of TEs and conflict between (sub)genomic elements within one nucleus (Parisod et al., 2012). Lim et al. (2004) suggested that greater imbalance is leading to stronger genome shock intensity. Recently, Mhiri et al. (2019) found that the proportion of new loci correlates with the extent of each TE load imbalance in different *Nicotiana* accessions and thus supported the influence of the genome shock intensity on TE activation. Genome dominance at the genomic level may be achieved by different regulation and composition of dominant TEs in the parental lineages (Freeling et al., 2012). The (re)activation of TEs includes re-patterning of DNA methylation in CG, CHG, and CHH motives, as well as changes in methylation of lysine residues in histone H3 which are typical for heterochromatin, namely, H3K9me2 and H3K27me1 or H3K27me2 (Lindroth et al., 2004; Fuchs and Schubert, 2012; Rando, 2012). Additionally, it is hypothesized that TE activity can contribute to genome size increase in a hybrid formation event. Conversely, unequal homologous recombination and illegitimate recombination may reduce the TE genome content (Bennetzen and Wang, 2014), as described for *Veju* elements in allohexaploid wheat (Kraitshtein et al., 2010). An extreme case of TE reactivation after interspecific hybridization may result in an increase of postzygotic lethality and seed abortion accompanied by arrested embryo development (Josefsson et al., 2006).

Newly resynthesized wheat, *Triticum aestivum* (with AABBDD genomes) represents a very good example of altered gene expression and altered DNA methylation affected by TEs activation after polyploidization. In this allopolyploid, the reactivated LTR *Wis 2-1A* retrotransposon deregulates expression of neighboring genes, driving the transcription of flanking regions (Kashkush et al., 2003). Similarly, the DNA methylation is altered in the case of *Veju* LTRs. About ∼43% of the tested insertion sites of *Veju* LTRs displayed hypo or hyper-methylation in the successive generations after allopolyploidization (Kraitshtein et al., 2010). In a later study, a similar heritable methylation repatterning was observed also for the *BARE-1* retroelement (Zhao et al., 2011). Given that both retroelements belong to Class I LTR (Wicker et al., 2007), it would be interesting to decipher how other retroelements and DNA transposons may affect the hybrid vigor in natural occurring hybrid populations. Interestingly, in naturally occurring wheat, the genome contains 2–10% less DNA than the sum of its putative diploid parents. The similar DNA elimination has been observed also in synthetic allopolyploids, showing such events can be studied in artificial system (Eilam et al., 2010). An intriguing question is how the parent-specific dominance of one genome can affect the properties of the newly formed hybrids and subsequent generations. In the allopolyploid *Nicotiana tabacum*, for example, the elimination of the paternally derived DNA was observed (Renny-Byfield et al., 2011).

Another unanswered question is how the alleles of paternal or maternal origin modulate the hybrids phenotype (vigor) and to which extent are paternal/maternal epigenetic mechanisms transmitted to the progeny. To answer such a complicated question, it will be necessary to study more hybrid systems and species (Song and Chen, 2015) and to formulate better hypotheses. We stress that high throughput sequencing methods now allow deeper understanding of the allopolyploidization process. Better understanding of the mechanisms underlying TE dynamics may allow development of new desired hybrids in near future.

## Genome and Nucleolar Dominance

The gene number (orthologs or homoeologs) is duplicated after allopolyploidization (two diploid genomes are merged into a tetraploid individual). As a result of the duplicated genes after allopolyploidization, the hybrid genomes undergo extensive changes in gene expression, called “transcriptomic shock.” This shock modifies the gene expression patterns, followed by unequal parental contribution and transgressive up- or down-regulation (Parisod et al., 2012; Yoo et al., 2013). From the long-term evolutionary perspective, there are three possible scenarios for ortholog genes (Figure 1B; Ma and Gustafson, 2005): (i) one copy becomes non-functional by genetic and/or epigenetic changes (non-functionalization), (ii) one copy acquires a novel, usually beneficial function, and is preserved by natural selection while the other copy retains the original function (neo-functionalization), or (iii) both copies become partially compromised by accumulations of mutations to the point where their total capacity is reduced to the level of the single-copy ancestral gene (sub-functionalization).

Parental genome which becomes dominant usually is the one that has lost fewer genes and therefore tends to express its genes to higher levels (Woodhouse et al., 2014). This phenomenon is called biased fractionation and is a result of functional conflicts between interacting genes and has been verified in many polyploid species (Emery et al., 2018). The dominance of a parental genome over the other genome at the gene expression level seems to be established in the first generations after hybridization and is transmitted over the generations (Schnable et al., 2011; Cheng et al., 2012; Zhu et al., 2017) or multiple rounds of polyploidy (Woodhouse et al., 2014). The genome dominance thus impairs the expression of various gene as demonstrated for rRNA genes (see below) and other genes, e.g., the genes for centromeric proteins. In fact, Talbert et al. (2002) used an antibody against *Arabidopsis thaliana* CenH3 and demonstrated that this antibody does not recognize the centromeres of *Arabidopsis arenosa* but recognizes the epitope in synthetic and natural allopolyploids originated from both these species. This clearly shows that the CenH3 gene from *A. thaliana* is dominant and its product is incorporated into the centromeres from both parental species. So, it seems that in a wide variety of hybrids one parent only recruits the kinetochore components (or its majority) which are expressed from the genes of the dominant parent. We hypothesize that the congruence between these components (kinetochore) and centromeric repeats probably determine the kinetochore function in newly formed hybrids and chromosome stability.

There are two different attributes which facilitate the genome dominance at the transcriptome level (Figure 1B), ELD, and homoeolog expression bias (HEB). While the ELD accounts for the overall expression of a single gene which resembles the expression level of one of its parents, the HEB represents preferential expression from one-parental allele (from one homoeolog) (Grover et al., 2012). Over the past decade, HEB and ELD were extensively studied in a number of plant allopolyploids (Table 1) (Bardil et al., 2011; Yoo et al., 2013; Edger et al., 2017; Wu et al., 2018). It should be stressed that majority of the genes can be expressed additively from both parental alleles even in cases with observed ELD and HEB (Chelaifa et al., 2013; Bertrand et al., 2015).

**TABLE 1:** Examples of genome dominance in different hybrids and polyploids.

Species	Genome dominance	Genomes	References
***Coffea arabica*** 2*n* = 4*x* = 44	ELD	*C. canephora* × *C. eugenioides*	Bardil et al., 2011
***Brassica napus*** (natural) 2*n* = 4*x* = 38, AACC	HEB	*B. rapa* × *B. oleracea*	Ksiazczyk et al., 2011; Chalhoub et al., 2014
***Brassica napus*** (resynthesized) 2*n* = 4*x* = 38, AACC	HEB, ELD	*B. rapa* × *B. oleracea*	Wu et al., 2018
***Triticum aestivum*** 2*n* = 6*x* = 42, BBAADD	HEB	*T. turgidum* × *A. tauschii*	Pfeifer et al., 2014; Harper et al., 2016; Ramirez-Gonzalez et al., 2018
***Triticum aestivum*** 2*n* = 6*x* = 42, BBAADD	ELD	AABB (*T. turgidum*) × DD (*A. tauschii*)	Li et al., 2014
***Mimulus peregrinus*** (natural and resynthesized), ***Mimulus robertsii*** (resynthesized triploid hybrid) 2*n* = 6*x* = 92, GGLLLL	ELD and HEB	*M. guttatus* × *M. luteus*	Edger et al., 2017
***Capsella bursa pastoris*** (*C. grandiflora* × *C. orientalis*) 2*n* = 4*x* = 32	ELD	*C. grandiflora* × *C. orientalis*	Kryvokhyzha et al., 2019
***Gossypium hirsutum*** 2*n* = 4*x* = 52	HEB, ELD	*G. arboreum* × *G. raimondii* (?)	Yoo et al., 2013

*ELD—expression level dominance, HEB—homoeolog expression bias.*

Generally, genome dominance is mediated by the upregulation of the dominant allele or downregulation of the submissive allele (Shi et al., 2012; Yoo et al., 2013; Combes et al., 2015; Zhu et al., 2017). If this is the case, then *trans* factors would likely be responsible for dominance, and the parent with more efficient *trans* factors would presumably be the one to establish the dominance (Hu and Wendel, 2019). Moreover, gene expression can be modified by epigenetic regulation mediated by TEs (see section “Genomic Changes and TE Dynamics in Allopolyploids”) and small RNAs (McClintock, 1984; Ha et al., 2009; Lu et al., 2012). Specifically, siRNAs serve as guides for methyltransferases to perform *de novo* DNA methylation at CG, CHG, and CHH motifs (Haag and Pikaard, 2011). The methylation level of these motifs is often modified (either increased or reduced) after hybridization (Greaves et al., 2015; Zhu et al., 2017). Methylation of TEs within or close to a gene can lead to its silencing (Kim and Zilberman, 2014; Rodrigues and Zilberman, 2015). As an example, higher siRNA density at genes associated with TEs showed a negative effect on gene expression of the D genome in nascent allohexaploid wheat (Li et al., 2014). In monkeyflower allopolyploids, the dominantly expressed genome displayed a lower abundance of TEs and a reduced level of the CHH site methylations near genes (Edger et al., 2017). However, the role of methylation at the CG, CHG, and CHH motifs may be meager. In general, 24nt siRNAs are downregulated in the hybrids at loci in which parents differ in sRNA levels. Additionally, it has been shown that closely related parental lines had more additive expression of 21nt miRNAs and hybrids formed from more divergent lines display several non-additively expressed miRNAs, altering gene expression and phenotype of F1 population. Thus, it is believed that competition of parental hybrids for TEs regulation and overall levels of siRNAs (Figure 1A) are important for the hybrid vigor, and hybrid expression changes (reviewed in Groszmann et al., 2013). A comparative study of 3DL chromosome arms from wheat and its progenitor *Aegilops tauschii* revealed that methylation is responsible for only 11% of genes with altered gene expression. It seems that a reduced gene expression correlates more tightly with higher compaction and reduced accessibility of chromatin of this particular wheat chromosome arm (Lu et al., 2019). This was not surprising because gene expression changes have been linked with the spatial organization of chromatin and gene repositioning (Lanctot et al., 2007; Gonzalez-Sandoval and Gasser, 2016). Recently, Wang et al. (2018) described the dynamics of 3D genome architecture after polyploidization, showing the reorganization of topologically associated domains in allopolyploid cotton. In addition, they identified inter-subgenomic chromatin interactions between homoeologous gene pairs. Linking these interactions with expression of homoeologous gene pairs showed that some genes with extreme expression bias are associated with low number of chromatin interactions. Increased compactness, however, did not correlate with gene expression changes in allotetraploid *Arabidopsis suecica* (a hybrid of *A. thaliana* × *A. arenosa*) (Zhu et al., 2017).

An important manifestation of the genome dominance in plant hybrids is nucleolar dominance (ND) first described by Navashin (1934). Nucleoli are sites of transcription of rRNA, which is participating in the ribosome assembly. A typical feature of ND is that ribosomal genes inherited from one (dominant) parental species are expressed and those inherited from the other parent are silenced (Neves et al., 1997; Lawrence et al., 2004; Idziak and Hasterok, 2008; Ksiazczyk et al., 2011; Borowska-Zuchowska et al., 2016). Expression analysis of the rRNA transcription in two yeast strains revealed that the proportion of active rDNA is regulated by a dosage control mechanism (French et al., 2003). The level of rDNA expression is about the same in both strains, even though they significantly differ in the number of copies. Such dosage control is a result of higher occupancy of Pol I per gene, and the occupancy itself is linked to epigenetic marks regulating it (French et al., 2003). Increased methylation of CHG and CHH motifs and histone marks (H3K27me3 and H3K9me2) was observed for A genome NOR (nucleolar organizing region) loci in synthetic allotetraploid wheat, leading to their silencing and further elimination in later generations (Guo and Han, 2014). Similarly, epigenetic modifications such as DNA and histone methylations at lysine residues (H3K9me2 and H3K4me3) resulted in the silencing of NORs of *A. thaliana* genome in allotetraploid *A. suecica* (Lawrence et al., 2004). Conversely, reduction of CG and CHG DNA methylation probably influenced the reactivation of NOR of the submissive genome in *Tragopogon mirus* (Dobesova et al., 2015). Similarly, the deletion of the NOR region to about 4% of the normal length does not significantly decrease the level of rDNA expression in *T. mirus* (Dobesova et al., 2015). The rDNA loci frequently differ in numbers between genomes in allopolyploids, e.g., in *Triticale*, one locus is present in the rye genome, while the wheat genome possesses two major and several minor loci. Interestingly, all rRNAs are transcribed from wheat loci (Neves et al., 1997) but not necessarily from rye. Similarly, allotetraploid *A. suecica* expresses rRNA from *A. arenosa* while the *A. thaliana* rDNA genes are silenced. However, a backcross of *A. suecica* to *A. thaliana* reverts such patterns, and the *A. arenosa* rDNA cluster becomes suppressed (Chen et al., 1998). One may expect that the ND is correlated with the number of rDNA loci of the parental genomes. Nevertheless, translocation of the short arm of rye chromosome containing nucleolus organizing regions (NORs) to its homoeologous wheat chromosome made the rye NOR co-dominant with wheat NORs (Vieira et al., 1990). Thus, the number of these loci itself is presumably not the exclusive driving force for the ND, and another mechanism operates. In fact, other studies support the hypothesis that selective silencing of rRNA genes depends on the position on the chromosome and sequences that surround them (Chandrasekhara et al., 2016; Mohannath et al., 2016). Yet, another factor in ND seems to be also parent-allele specific origin. In allopolyploids such as *Tragopogon* L., *Cardamine* L., and *Senecio* L., NOR is preferentially expressed from the maternal genome. On the other hand, some hybrids display a bias in the expression of rRNA genes toward the same genome in reciprocal crosses. In hybrid *Rosa agrestis* and *Rosa rubiginosa*, the expression dominance of the *Canina* type units was observed even if they were underrepresented in copy numbers (Khaitová et al., 2010; Herklotz et al., 2018). Overall, in some systems, one-genome-type rDNA is more vulnerable to repression, and in others, it is prone to be dominant. Such vulnerability is clearly linked with epigenetic marks (Costa-Nunes and Pontes, 2013; He and Deng, 2013). Nevertheless, it remains to be determined how such ND is established, and whether one parent always becomes dominant or factors such as neighbor regulatory sequences, chromosomes positioning, and chromatin organization during interphase play a role.

## Chromosome Pairing in Hybrids

Homoploid hybrids are rare in plants (Yakimowski and Rieseberg, 2014). This may well be due to problems in meiosis when homoeologous chromosomes fail to pair as bivalents, and random segregation of univalents produces non-functional gametes. Thus, WGD of sterile diploid F_1_ hybrids is necessary for fertility restoration.

The homoploid hybrids occur in nature only sporadically. For this reason, we focus here on allopolyploids and provide only several known examples of chromosome rearrangements in artificial homoploid hybrids. Allopolyploid hybrids possess three or more chromosome sets from two or more species, e.g., *A. suecica* (Novikova et al., 2017). Assuming that the basic chromosome number is the same in both parents, each chromosome in an allopolyploid can pair either with its homolog or with one of the two homoeologs. Theoretically, in an allotetraploid, the ratio of homologous vs. homoeologous pairing should be 1:2, but very few hybrids exhibit such a ratio. The pairing bias depends on the level of the DNA sequence divergence of two parental genomes. Immediately or soon after initial hybridization, newly formed allopolyploid lineages often establish a system that may hamper the pairing of homoeologs. One of the such best-studied systems is *Ph1* (Pairing homoeologous 1) presented in polyploid wheats (Sears and Okamoto, 1958). Nevertheless, after 60 years of extensive research, the mode of action of *Ph1* is still not completely understood. There are two competing theories to its actual location and nature (Rey et al., 2017, 2018; Rawale et al., 2019). It is assumed that the *Ph1* locus contains a cluster of defective cyclin-dependent kinases (CDKs) and S-adenosyl methionine-dependent methyltransferase (SAM-MTases) genes and inserted paralog of the ZIP4 (Knight et al., 2010; Greer et al., 2012; Martin et al., 2014, 2017). Interestingly, *Ph1*, once introgressed from wheat to the relative species, has an ability to modify chromosome pairing of the host genome (Lukaszewski and Kopecky, 2010). Coupled with *Ph1*, a similar chromosome pairing control systems have been found in *Brassica* allopolyploids (*PrBn*), oats, fescues, and many other allopolyploids (Jenczewski and Alix, 2004). Nevertheless, the presence of a regulating system does not always preclude the elimination of the submissive genome as documented in triticales (allopolyploids of bread wheat and rye) (Tsunewaki, 1964; Orellana et al., 1984; Lukaszewski et al., 1987).

The mechanism of reduced chromosome pairing in allopolyploids is also not fully understood. In wheat-rye hybrids, a relationship between the behavior of telomeres and the success of chromosome pairing has been observed by Naranjo (2014), who reported that reduced pairing of rye chromosomes in wheat appeared to be a consequence of disturbed migration of rye telomeres into the leptotene bouquet. We have observed that the problem of aberrant rye telomeres is not limited to the initial stages of meiosis but may be systemic in nature. The frequency of out-of-bouquet rye telomere positions at leptotene was virtually identical to that in the nuclei of somatic cells, and that in turn correspond to the rate of chromosome pairing (Pernickova et al., 2019a, b).

Besides allopolyploids with evolved pairing regulators, several hybrids with extensive homoeologous chromosome pairing have been reported in well-established allopolyploids and synthetic F_1_ hybrids (either homoploids or allopolyploids). Some allopolyploids with a molecular mechanism of diploid-like pairing behavior, when resynthesized from putative progenitors, display disrupted meiosis with homoeologous chromosomes pairing as described in resynthesized *Brassica napus* (Gaeta and Pires, 2010). Xiong et al. (2011) studied the karyotypes of resynthesized *B. napus* and found that the aneuploidy rate was increasing for ten successive generations. In addition, the authors found frequent homoeologous chromosome pairing and replacement of chromosomes of one parental species by the other (prevalence of C-genome). Intriguingly, two lines retaining the expected original chromosome constitution had the highest seed yield, and thus, the selection against aberrant chromosome constitutions with reduced fertility may be expected. Those lines which lack the control over the regular meiotic division might be the key factor in the establishment of natural *B. napus* with the stabilized genome.

## Parental Chromosome Dominance

The mechanism(s) responsible for the chromosomal genome dominance remains to be fully determined. There are several features that may hypothetically facilitate the replacement of chromosomes from the submissive genome by those from the dominant genome (Figure 1C). Possible scenarios involve differences in male meiosis, female meiotic drive, variation in the proliferation of pollen tube, germination, and fertility of pollen grains and seed yield. A combination of these processes is likely involved; however, technical issues hamper the ability to discriminate among individual factors clearly. The submissive genome may show reduced chromosome pairing; this leads to the formation of univalents, and univalents are frequently lost during meiosis. However, the substitution of chromosomes from one genome by homoeologs chromosomes from the other, and not just uniparental chromosome elimination, is far more complicated. Such chromosomal substitution would require non-disjunction and unidirectional movement of both homologs from the dominant genome (associated in the bivalent) to one pole of the meiotic cell (coupled with concomitant elimination of the homoeolog). This does not appear very likely. Random migration of univalents to daughter nuclei in anaphase I during meiosis would probably be more likely to produce such single chromosome substitutions, coupled with selection for euploid gametes.

In our earlier studies, we have observed chromosomal dominance in all cultivars selected from *Lolium multiflorum* × *Festuca pratensis* hybrids. These hybrids exhibit the prevalence of the *Lolium* chromosomes (Kopecky et al., 2006). Zwierzykowski et al. (2006, 2011) conducted a study over eight successive generations of such hybrids and observed a slow but consistent gradual replacement of the *Festuca* by *Lolium* chromosomes. Such chromosome-level genome dominance appears to be present also in other hybrid systems. In homoploid onion hybrids of *Allium cepa* × *Allium roylei*, the *A. roylei* genome appears to replace the *A. cepa* genome in successive generations. van Heusden et al. (2000) reported that, on average, the *A. cepa*-specific markers were not amplified in 28% of the F_2_ plants, while only 16% of the F_2_ plants did not display amplification of *A. roylei*-specific markers. Thus, the contribution of *A. cepa* and *A. roylei* alleles in the F_2_ population was 44 and 56%. Similar genome dominance on the chromosome level has been observed in hybrids of *Alstroemeria aurea* × *Alstroemeria inodora*, *Gasteria lutzii* × *Aloe aristata* and in various lily hybrids (Takahashi et al., 1997; Kamstra et al., 1999; Karlov et al., 1999; Khan et al., 2009).

The role of meiotic pairing in chromosomal genome dominance is speculative. In an F_1_ hybrid originating from crossing autotetraploid *L. multiflorum* and allotetraploid *Ferocactus glaucescens*, we observed 101 univalents of the former and 161 univalents of the latter (Kopecky et al., 2009). On contrary, in tetraploid hybrids *Lolium perenne* × *Formica pratensis*, Zwierzykowski et al. (2008) found that *Lolium* univalents were more frequent. Thus, the role of male gametic selection does not seem to play a significant role. In contrast, asymmetrical female meiosis offers an unprecedented opportunity to modify the genomic composition in favor of the dominant genome. Far from plant hybrids, Akera et al. (2017) observed uneven positioning of the parental chromosomes on the meiotic spindle in hybrid mice. The positioning of chromosomes from the dominant genome toward the egg cell and of chromosomes from the submissive genome toward the polar body was more frequent than the reciprocal configuration. In female meiosis, only one of the four products forms the embryo sack and is transmitted to the next generation. Given that only one product of meiosis proceeds, preferential positioning of specific chromosomes in the first meiotic division can easily explain different rates of chromosome transmission. Shifts in proportions of the parental genomes in favor of the dominant one. The candidate molecular driver in this case is CDC42, which is signaling unequal regulation of microtubule tyrosination. This unequal tyrosination is probably caused by the difference in the copy number of kinetochores between the two genomes. Hence, the dominant genome (having more copies of centromeric repeats and kinetochore proteins) is preferentially transmitted to egg cell while the submissive one into the polar body (Chmatal et al., 2014).

## Discussion and Outlook

Our knowledge about the genome dominance and its consequences for the structure and evolution of hybrid genomes is still limited, and further studies are required to shed more light on this biologically intriguing phenomenon. A systematic and complex approach should be applied to several systems to uncover potential linkages among different attributes and their specific roles in genome dominance. In some plant systems, one genome dominates on all the possible levels such as seen in *Lolium* × *Festuca* hybrids: *Lolium* chromosomes predominate in consecutive generations (Zwierzykowski et al., 2006), *Lolium* alleles are overexpressed relative to those of *Festuca* (Stoces et al., 2016), seed yield is higher in plants with higher proportions of *Lolium* chromatin (Kubota et al., 2019), and rRNA is exclusively transcribed from the *Lolium* variant (Mahelka and Kopecky, unpublished). On the other hand, analyses of other allopolyploids often produce opposite or conflicting results. Perhaps the patterns of interactions in individual hybrids are specific to combinations of involved parents and allelic variants individual hybrids.

The fate of newly established allopolyploids and the dominance of one genome over the other is, so far, not fully predictable. Studies on *Brassica* indicate that lines with additive karyotypes, without any rearrangements, are the most fertile. Hence, they can be considered evolutionarily the most successful. In the situation of competition between the genomes, one could reasonably expect that the dominant genome would trigger elimination (or replacement) of the other genome and revert the hybrid to the parental form. In this situation, it would be in the best interest of the submissive genome to establish as quickly as possible a mechanism favoring homologous pairing over that of homoeologous. Consequently, if fertility (seed set and seed yield) is highest in the additive karyotype (both parental genomes present), it would be beneficial to establish control pairing mechanism for both genomes. In *B. napus*, an amphiploid of *Brassica rapa* (A genome) and *Brassica oleracea* (C genome), meiotic regulator *PrBn* is located on the linkage group 15 in genome C, the genome that clearly dominates on the chromosome and transcriptome levels in the first generations of resynthesized lines (Liu et al., 2006). In wheat, the B genome carrying the *Ph1* locus on its 5BL chromosome arm expresses its alleles at the same or very similar level as the other two genomes (Ramirez-Gonzalez et al., 2018). Although *PrBn* and *Ph1* models are so far only two best-known examples, further analyses are needed to shed more light on these phenomena. Another interesting question is if and how is regulated meiotic pairing in gymnosperms and gymnosperm hybrids (Sedel’nikova et al., 2011).

We stress that with an increased understanding of the principles and mechanisms of genome dominance in interspecific hybrids, it may be possible to predict which crosses will result in stable introgression of desired traits in plant breeding. It is still largely unknown if the genome dominance is fully deterministic or if it can be manipulated by the external conditions. Detailed molecular analyses of many model species and a complex view are needed to understand fully the complex aspects of hybrid formation and polyploidy evolution. We expect that future implication of CRISPR/Cas9 technology to edit methylation states, TE insertions, or species-specific centromere binding proteins can lead to the development of stable hybrids without presumable suppression of one of the genomes.

## Author Contributions

MG, VB, RH, and DK wrote the first draft of the manuscript. All authors contributed to manuscript revision and read and approved the submitted version.

## Conflict of Interest

The authors declare that the research was conducted in the absence of any commercial or financial relationships that could be construed as a potential conflict of interest.
